# Effect of Copper and Zinc on the Single Molecule Self-Affinity of Alzheimer’s Amyloid-β Peptides

**DOI:** 10.1371/journal.pone.0147488

**Published:** 2016-01-25

**Authors:** Francis T. Hane, Reid Hayes, Brenda Y. Lee, Zoya Leonenko

**Affiliations:** 1 Department of Biology, University of Waterloo, Waterloo, Ontario, N2L 3G1, Canada; 2 Department of Physics and Astronomy, University of Waterloo, Waterloo, Ontario, N2L 3G1, Canada; Russian Academy of Sciences, Institute for Biological Instrumentation, RUSSIAN FEDERATION

## Abstract

The presence of trace concentrations of metallic ions, such as copper and zinc, has previously been shown to drastically increase the aggregation rate and neurotoxicity of amyloid-β (Aβ), the peptide implicated in Alzheimer’s disease (AD). The mechanism of why copper and zinc accelerate Aβ aggregation is poorly understood. In this work, we use single molecule force spectroscopy (SMFS) to probe the kinetic and thermodynamic parameters (dissociation constant, K_d_, kinetic dissociation rate, k_off_, and free energy, ΔG) of the dissociation of an Aβ dimer, the amyloid species which initiates the amyloid cascade. Our results show that nanomolar concentrations of copper do not change the single molecule affinity of Aβ to another Aβ peptide in a statistically significant way, while nanomolar concentrations of zinc decrease the affinity of Aβ-Aβ by an order of magnitude. This suggests that the binding of zinc ion to Aβ may interfere with the binding of Aβ-Aβ, leading to a lower self-affinity.

## Introduction

We characterized the effect of trace concentrations of copper (Cu^2+^) and zinc (Zn^2+^) ions on the dissociation constant, K_d_, kinetic dissociation rate, k_off_, and the difference in free energy between the bound and unbound states, ΔG, of the dissociation of two amyloid-β (1–42) (Aβ42) monomers forming an amyloid dimer, the smallest neurotoxic species implicated in Alzheimer’s disease (AD) [1]. The dissociation constant, K_d_ is a common measure of chemical affinity. A lower K_d_ is indicative of a higher chemical affinity [2]. Trace concentrations of metal ions such as Cu^2+^and Zn^2+^ have been implicated in increased aggregation and neurotoxicity of Aβ42, accelerating the pathogenesis of AD [3]. The effect of metal ions on the dimerization of two Aβ peptides, the initial step in the amyloid cascade, is necessary to fully characterize the mechanism of how metal ions contribute to the pathogenesis of AD.

AD is a neurodegenerative disease believed to be caused by the aggregation of the Aβ peptide into toxic oligomers [4–6]. These toxic oligomers can take the form of a variety of different morphologies including prefibrillar oligomers, amyloid derived diffusible ligands and annular protofibrils [7]. The first initial step in the formation of these oligomers and fibrils is binding of two monomeric amyloid peptides together to form a dimer, which serves as a nucleation unit for further oligomerization and fibrillization. These dimers are less toxic than tetramers and trimers, but more toxic than fibrils and monomers [1].

Extensive research has demonstrated that metal ions accelerate the effects of Aβ aggregation [8–14], diverting the amyloid cascade along different pathways and making amyloid clearance more problematic [10]. In addition to these kinetic effects, the redox effects of metal ions contribute to neurotoxicity via lipid peroxidation [6, 9].

The aggregation cascade of Aβ begins with an Aβ monomer misfolding into an internal β-sheet with the amino acids 17–23 binding to 28–35 and the amino acids 24–27 forming a hinge-like β-hairpin to allow the peptide to fold back on itself [15]. The cascade continues with the monomer folding into a dimer, the smallest neurotoxic amyloid species [6, 16]. The peptide can then travel along a variety of different reaction pathways leading to stable structures such as oligomers and mature fibrils [10].

The role of metal ions on amyloid aggregation and toxicity has been studied extensively [9, 17]. Amyloid plaques have been shown to contain higher than physiological levels of Cu^2+^ and Zn^2+^ ions [18, 19]. Cu^2+^ ions increase neurotoxicity by both accelerating amyloid aggregation and increasing oxidative stress [9, 20]. This increased propensity of Aβ to aggregate in a Cu^2+^ rich environment has been attributed to Cu^2+^ ions causing Aβ to move closer to its isoelectric point, thereby inducing aggregation [13]. By contrast, Zn^2+^ ions appear to have a mixed effect on Aβ toxicity: some reports have noted a neurotoxic effect while others have demonstrated a neuroprotective effect of Zn^2+^ ions [21, 22]. This discrepancy may be the result of different concentrations of Zn^2+^ studied in between reports [23]. Increased concentrations of metal ions in AD patients is believed to be caused by dyshomeostasis as opposed to excess dietary intake since copper is regulated by the blood-brain barrier (BBB) [24, 25]. Aβ contains a metal binding site whereby the imidazole group of His6 from one monomer coordinates with the imidazole groups of His13 and His 14 residue of another monomer, mediated by a copper or zinc ion [22, 26–28]. This Cu^2+^-Aβ affinity has been shown to be in the nanomolar to picomolar range [12, 29, 30]. However, our work is the first report calculating the affinity of two Aβ peptides *to one another* in the presence of nanomolar concentrations of Cu^2+^ or Zn^2+^ ions.

In our earlier work we showed that Cu^2+^ ions increased the unbinding force between two Aβ (1–42) peptides [11]. Abnormal amyloid clearance has been demonstrated to be the leading mechanism contributing to late onset AD, i.e. persons without familial early onset AD [31]. Our previous results led us to hypothesize that since early onset AD is largely a function of abnormal Aβ clearance, increased affinity of Aβ would make amyloid clearance considerably more problematic. In this work we used single molecule force spectroscopy (SMFS) [32] to further elucidate the mechanism of the very initial stage of amyloid aggregation—the dimerization of two peptides. SMFS is a nanoscale technique whereby an atomic force microscopy (AFM) tip is used to probe the unbinding of two molecules of interest—one bound to the surface, the other to the tip. When AFM tip is brought to the surface the binding between two Aβ monomers occur and when the tip is retracted the unbinding forces between two monomers are measured. To extract kinetic and thermodynamic parameters governing the system, the retraction velocity of the tip is varied. This iteration of SMFS is referred to as dynamic force spectroscopy (DFS) [33].

The application of Friddle-De Yoreo reversible binding model to the DFS data allows one to extract kinetic and thermodynamic parameters, such as dissociation constant, K_d_, kinetic dissociation rate, k_off_, and free energy, ΔG [34, 35].

## Methods

### Surface Preparation and Atomic Force Spectroscopy

We prepared our experiments in accordance with our previous force spectroscopy experiments [11, 35, 36], of which the methods are detailed extensively in [23]. Briefly, Veeco MLCT SiNi AFM cantilever were cleaned by soaking in ethanol and exposing to UV light for 30 minutes. Freshly cleaved mica and the AFM cantilevers were soaked for 30 minutes in 167 μM aminopropylsilatrane (APS) to silanate the surface. The mica and cantilevers were then soaked for 3 hours in a solution of 3400 MW NHS-PEG-MAL (Laysan Bio, Alabaster GA) solution. A solution of 20 nM cys-amyloid-β (1–42) (rPeptide, Bogart USA) was prepared by dissolving 0.5 mg cys-Aβ42 in 1 mL of DMSO followed by serial dilutions in HEPES 50 mM buffer (pH 7.4, 150 mM NaCl) to achieve the final concentration. The surface of the cantilever tip and mica surface were soaked in the Aβ solution for 30 minutes. Following rinsing, unreacted maleimide groups were quenched with b-merceptoethanol. Tips and mica were then rinsed and stored in HEPES buffer.

Typical cantilever spring constants were between 8 and 120 mN/m but varied with each individual experiment. Spring constants were measured using Hutter’s thermal noise method [37]. A series of force curves were taken with an approach and retract velocity varying between 50 and 10,000 nm/s resulting in loading rates between 0.3 and 300 nN/s. Following the collection of an Aβ42 data set in HEPES buffer, a 20 nM metal ionic solution (Cu^2+^ or Zn^2+^) in HEPES buffer was added to the liquid cell of the AFM and additional force curves were collected for comparison.

### Force Curve Analysis

JPK data analysis software was used to analyze force curves. We selected force curves for further analysis which had a contour length between 40–150 nm and would fit a worm-like chain (WLC). The force curves were smoothed, the x-axis leveled, both axes set to 0, and the force curve corrected for cantilever bending to obtain tip-sample separation, z. A WLC fit was obtained for each force curve while rupture force and contour length data were recorded. Data was sorted in order of rupture force and the highest rupture force data were eliminated using Poisson statistics to eliminate force curves that were likely the result of simultaneous multiple unbinding events [38].

### Extraction of Kinetic Data–Friddle-De Yoreo Model

Loading rates were corrected to account for the effect of the PEG linker [39]. We fit the Friddle-De Yoreo reversible binding equation to the entirety of the dataset (comprised of the rupture force and corrected loading rate of each force curve) using a Levenberg-Marquardt best fit algorithm in Origin 9.0 graphing software. For pooled datasets, we used the mean spring constant of the cantilevers used in the experiments. We extracted the equilibrium force, f_eq_, the thermal scaling factor, f_β_, and the kinetic off rate, k_off_(f_eq_) at the equilibrium force as well as standard errors from the dataset using the graphing software. These parameters were then used to calculate parameters ΔG (the difference in free energy between the bound and free states), x_β_ (the width of the energy barrier), k_off_ (the kinetic dissociation, or off- rate), and K_d_ (the dissociation constant) using the equations used in [34, 35].

### Statistical Analysis

Analysis of variance (ANOVA) testing was conducted on ΔG, x_β_, k_off_, and K_d_. Following the ANOVA test, a *post hoc* Welsh’s t-test was conducted to compare the parameters for the aqueous experiments compared to the copper experiments and for the aqueous environment experiments compared to the zinc experiments.

## Results and Discussion

We collected rupture force versus loading rate data using DFS. We used the Friddle-De Yoreo model [34] model to extract kinetic and thermodynamic information about the unbinding of an Aβ dimer in the presence of nanomolar concentrations of Cu^2+^ and Zn^2+^ ions.

**Fig 1:**
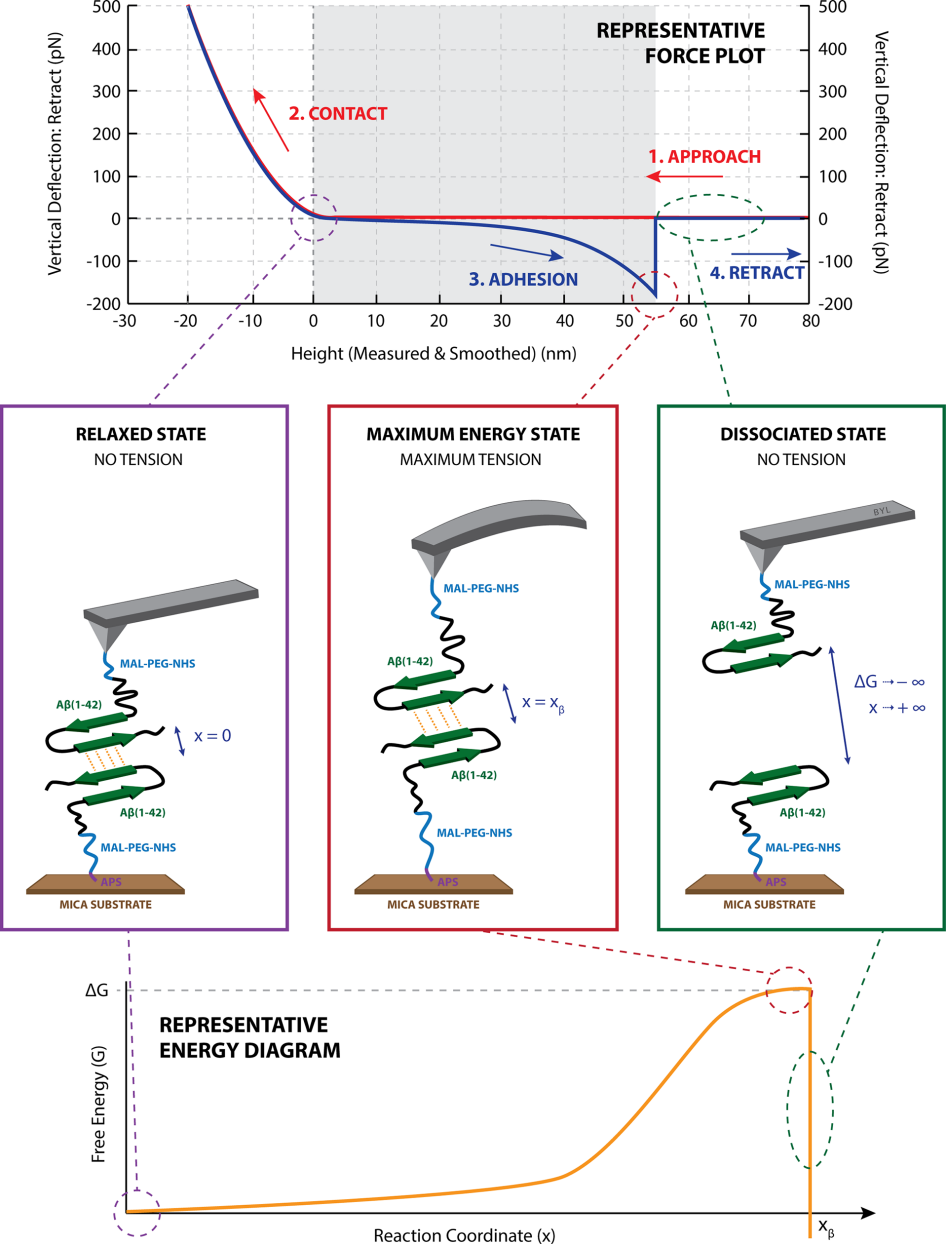
Mechanically induced Aβ binding and dissociation. (A) is a representative experimental force plot: the tip approaches the sample (red line) and when it touches the surface—the cantilever bends (steep linear region). The two surface-bound monomers are allowed to bind (red to blue transition at top of linear region. The cantilever retracts (blue steep linear region). As the cantilever returns to its neutral position, the force plot passes through the base line. At this point the system is in its minimum free energy state (B). The cantilever is not deflected and the system resembles a stable dimeric state (B). As the cantilever retracts, a mechanical force is applied along the reaction coordinate and the free energy of the system increases (E). The system reaches its maximum free energy just prior to rupture at x = x_β_ (E) at which point the cantilever is at its maximum deflection (C). At dissociation, the cantilever returns to its neutral position (D) and all free energy of the system is lost [40].

During an SMFS experiment, one molecule is tethered to the substrate and the other to the tip via a heterobifunctional cross linker (such as NHS-PEG-maleimide) (Fig 1). As the two monomers are mechanically dissociated at various loading rates (dF/dt), the dissociation rate (k_off_) increases exponentially to the natural logarithm of the rupture force, while the association rate (k_on_) decreases with increasing rupture force [34]. The force at which k_off_ and k_on_ are equal is referred to as the equilibrium force, f_eq_, at which the system transitions from the equilibrium regime to the kinetic regime.

The equilibrium force is given by the equilibrium equation [34],

$$f_{eq}=\sqrt{2k_{c}\Delta G}$$ Eq. 1

Where k_c_ is the spring constant of the system and ΔG the difference in free energy between the bound and unbound states.

The mean unbinding force, ‹F(r)›, is approximated by the equation [34],

$$\langle F(r)\rangle ≅f_{eq}+\;f_{\beta }\;\text{ln}\;\left(1+\;\frac{{re}^{-\gamma }}{k_{off}\left(f_{eq}\right)f_{\beta }}\right)$$ Eq. 2

Where γ is Euler’s constant, 0.577. The thermal force scale, f_β_, is given by the expression f_β_ = k_B_T/x_β_. k_off_(f_eq_) is the dissociation rate at the equilibrium force, f_eq_. ‹F(r)› is the mean rupture force as a function of the loading rate, r, corrected for the effect of the PEG linkers as explained in [39].

From f_β_, the width of the energy barrier, x_β,_ can be calculated by the equation [34],

$$x_{\beta }=\frac{k_{B}T}{f_{\beta }}$$ Eq. 3

The dissociation rate at force F, k_off_ (F), is given by the function [34],

$$k_{off}(F)=k_{0}\;\text{exp}[\beta \left(Fx_{\beta }-1/2k_{c}{x_{\beta }}^{2}\right)]$$ Eq. 4

The association rate (on rate), k_on_(F), is given by the function [34],

$$\begin{array}{l} k_{on}(F)=k_{on}(0)\;\text{exp}\;\left[-\frac{\beta k_{c}}{2}{\left(\frac{F}{k_{c}}-x_{\beta }\right)}^{2}\right]\\ k_{on}(F)=k_{off}(F)\;\text{exp}\;\left[\beta \;\left(\Delta G-\frac{F^{2}}{2k_{c}}\right)\right] \end{array}$$ Eq. 5

The dissociation constant, K_d_, is a measure of the chemical affinity of two molecules. A lower dissociation constant corresponds to a higher chemical affinity.

Eqs 4 and 5 can be reduced to,

$${e^{k_{B}T\Delta G}}^{-1}=\frac{k_{on}}{k_{o\;ff}}={K_{d}}^{-1}$$ Eq. 6

**Fig 2:**
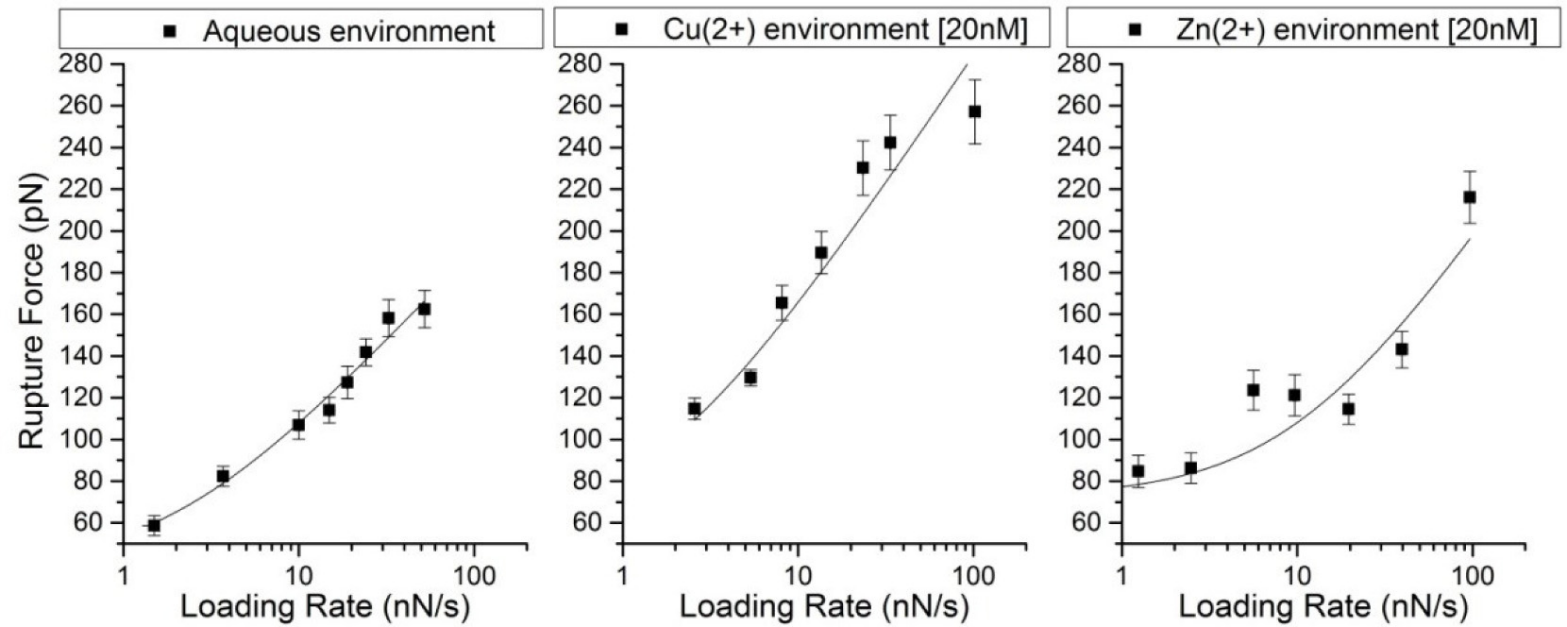
Force vs loading rate plots for Aβ42 dimer dissociation in aqueous, Cu^2+^ [20nM], and Zn^2+^ [20nM] environments. The force plots have been fit with the Friddle-De Yoreo reversible binding model [34]. Adjusted R^2^ values of fits are 0.979, 0.897, and 0.808 for aqueous, copper, and zinc data, respectively.

Plotting these data points to create a force spectrum (Fig 2) and fitting Eq 2 to these data points, we calculated kinetic and thermodynamic properties shown in Table 1. We calculated a chemical dissociation constant, K_d_, of 4.02×10^−4^ M for our control experiment of Aβ dimerization in HEPES buffer compared to 1.73×10^−4^ M for Aβ with 20 nM Cu^2+^ and 7.50×10^−3^ M for Aβ with 20 nM Zn^2+^.

**Table 1:** Kinetic and thermodynamic parameters for Aβ-Aβ unbinding in aqueous buffer, Cu^2+^ [20nM] and Zn^2+^ [20nM] solutions.

	ΔG (k_B_T)	k_off_ (s^-1^)	k_on_ (M^-1^s^-1^)	K_d_ (mM)
**Aβ-Aβ in aqueous buffer**	7.83 ± 3.03	12.5 ± 9.62	31250 ± 27140	0.40± 0.16
**Aβ-Aβ with Cu**^**2+**^ **[20nM]**	8.66 ± 1.62	23.4 ± 9.72	137640 ± 62119	0.17 ± 0.03
**Aβ-Aβ with Zn**^**2+**^ **[20nM]**	4.89 ± 1.35	57.3 ± 56.30	7640 ± 7794	7.50 ± 2.06

A number of groups have calculated rate constants and free energies of amyloid monomers binding to and elongating amyloid fibrils [41, 42]. While these works are remarkable, our work is the first to measure the free energy and dissociation constants of an Aβ dimer on a single molecule level.

Cannon *et al*. used surface plasmon resonance (SPR) to calculate reaction rates for fibril elongation [42]. While this elongation reaction occurs further along the amyloid cascade than in our work, it does serve as a useful comparison for our experiments. Using their published association (k_1_) and dissociation rates (k_-1_), we calculated a dissociation constant (k_-1_/k_1_) of K_d_ = 1.23×10^−4^ for a monomer-fibril complex. By comparison, we calculated a dissociation constant between two amyloid monomers forming a dimer of K_d_ = 4.02×10^−4^. While these dissociation constants are within the same order of magnitude we attribute the difference to the difference between the monomer-monomer binding mechanism in our study and the monomer-fibril mechanism in the report by Cannon et al. The monomer-fibril binding mechanism is possibly affected by the cross-linking of the additional monomer already incorporated in the amyloid fibril.

Buell *et al*. used quartz crystal microbalance (QCM) to measure thermodynamic parameters of fibril elongation [41]. The energy barrier of fibril elongation is higher than that for amyloid dimerization. The activation energy of Aβ42 fibril elongation was calculated to be 2.42 k_B_T (5.9 kJ/mol). We calculated a free energy of activation of 7.83 k_B_T for the Aβ dimer formation using SMFS, which is larger than that reported for fibril elongation suing QCM. Our calculated free energy of activation correlates well with our previous report [35].

To further understand the effect of metal ions on amyloid aggregation, we studied the effect of nanomolar concentrations of Cu^2+^ and Zn^2+^ ions, an environment roughly analogous to the environment resulting from metal dyshomeostasis which occurs in AD patients. Previous reports have shown that even nanomolar concentrations of copper and zinc ions may affect the aggregation and toxicity of Aβ [43].

Copper ions have a very strong affinity for Aβ in the nano- to picomolar range [12, 29, 30]. In this work, rather than determining the affinity of metal ions to the Aβ monomer, we calculated the self-affinity of two Aβ42 peptides in the *presence* of copper and zinc ions. We observed that the presence of zinc ions increases the dimer dissociation constant (which corresponds to a decreased monomer-monomer chemical affinity) by an order of magnitude (Table 1), whereas the presence of copper ions does not change the dissociation constant by a statistically significant amount.

Pedersen *et al*. who demonstrated that with trace concentrations of copper, Aβ begins to aggregate instantly compared to the characteristic lag period observed in aqueous environments without metal ions present [44]. Similarly, Sarell and colleagues observed that substoichiometric concentrations of Cu^2+^ ions induce acceleration in both the nucleation and elongation phases of amyloid fibril formation [13]. Sarell suggested that Aβ-Cu^2+^ binding at physiological pH caused Aβ to approach its isoelectric point inducing self-association and fibril formation. Despite the well established effect of copper on aggregation rates of Aβ, we did not measure a significant effect of copper ions on amyloid-amyloid affinity on a single molecule level. This could be due to the fact that effect of Cu on may not be significant on a single molecule level during Aβ dimerization, but may affect the aggregation rate of Aβ at later stages, past dimer formation.

We calculated association rates (k_on_) for each experiment using the relation k_on_ = k_off_/K_d_. Curiously, we calculated differences in k_on_ of approximately half an order of magnitude higher in the Cu^2+^experiment compared to the aqueous control and half an order of magnitude lower in the Zn^2+^experiment (Table 1). The association rate of two proteins is affected by a number of factors including ionic strengths which influence the electrostatic interactions and hydrophobic interactions [45]. Long range electrostatic interactions are associated with higher association rates. Our experiments revealed an association rate in the Cu^2+^ experiments approximately five times higher than the association rate in the control experiments. We infer that the ion-induced electrostatic interactions mediated by the Cu^2+^ ions in the solution result in this higher observed association rate. Conversely, we observed an association rate one-fifth of the control experiment in our Zn^2+^ experiment. Lower association rates indicate an absence of intermolecular forces and diffusion controlled association [45]. Due to the different amyloid coordination between Cu^2+^ and Zn^2+^, we speculate that Zn^2+^ ameliorates the electrostatic and hydrophobic forces associated with amyloid binding and the association is almost strictly diffusion controlled.

Mawuenyega and colleagues demonstrated that abnormal amyloid levels in the brain of late onset AD patients is caused by insufficient clearance of Aβ as opposed to the over production of Aβ42 which is the case in early onset AD [31]. Metal ions have been demonstrated to accelerate amyloid aggregation both *in vivo* and *in vitro* [13, 46]. Together with our previous work [35], we hypothesized that the presence of nanomolar concentrations of Cu^2+^ and Zn^2+^ would increase the affinity of Aβ monomers to one another forming an Aβ dimer. Despite Cu^2+^ increasing the affinity of Aβ-Aβ by a factor of two, this difference in affinity was insufficient to be statistically significant (p = 0.72). By contrast, our data showed that the presence of Zn^2+^ decreased the height of the energy barrier and increased the affinity (K_d_) of Aβ-Aβ from 0.40 ± 0.16 mM without Zn to 7.50 ± 2.06 mM with Zn ions present, (p = 0.013). Aβ- Zn^2+^ coordination has been well characterized and we expected that given the covalent nature of the Aβ- Zn^2+^ bond, the chemical affinity between two Aβ monomers in the presence of Zn^2+^ would be considerably higher than the affinity in a zinc-free environment. This suggests that the binding of the Zn^2+^ to Aβ may interfere with the binding of Aβ-Aβ leading to a lower self-affinity in Zn^2+^ environments.

We hypothesized a correlation between previously reported amyloid-β aggregation rates and single molecule chemical affinity. Our observations did not support this hypothesis. We speculate that initial single molecule binding of two monomers is not a fastest stage in aggregation rates of amyloid-β. Alternatively, it is plausible that chemical affinity, on a much larger scale than the single molecule level which we measured in this work, may influence the aggregation rate. For example, the chemical affinity of amyloid oligomers may have a much greater contribution on aggregation rates than the affinity of two amyloid-β monomers. We showed that Zn and Cu ions have different effects on a single molecule monomer-monomer interaction.

## Conclusion

Using dynamic force spectroscopy, we determined the chemical affinity of two Aβ monomers forming an amyloid dimer on a single molecule level and compared the effect of nanomolar concentrations of copper and zinc ions on the chemical affinity of Aβ. Applying Friddle-De Yoreo reversible binding model we extracted dissociation constant, K_d_, kinetic dissociation rate, k_off_, and free energy, ΔG, to elucidate the effects of a copper and zinc ions. Our results demonstrate that a nanomolar concentration of Cu^2+^ does not change the single molecule affinity of Aβ-Aβ and a nanomolar concentration of Zn^2+^ decreases the affinity of Aβ-Aβ interactions by an order of magnitude.
